# Repeat infection with Chlamydia trachomatis: a prospective cohort study from an STI-clinic in Stockholm

**DOI:** 10.1186/1471-2458-9-198

**Published:** 2009-06-22

**Authors:** Karin Edgardh, Sharon Kühlmann-Berenzon, Maria Grünewald, Maria Rotzen-Östlund, Ivar Qvarnström, Jennie Everljung

**Affiliations:** 1 Dept of Obstetrics and Gynecology, Sesam City, Karolinska University Hospital Solna, Stockholm, Sweden; 2 Dept of Epidemiology, Swedish Institute for Infectious Disease Control, Nobels väg 18, 171 82 Solna, Sweden; 3 Dept of Clinical Microbiology, Karolinska Institute/Karolinska University Hospital Solna, Stockholm, Sweden

## Abstract

**Background:**

Infection with genital Chlamydia trachomatis (Ct) is the most common notifiable sexually transmitted infection (STI) in Sweden. A mutated Chlamydia, nvCT, has contributed to the increase. The occurrence of repeat infections is not investigated in Sweden. The current paper presents the study protocol for the first Swedish clinical investigation of repeat Chlamydial infection. The concern of the study is whether a Chlamydia infection at inclusion indicates an increased risk for Chlamydia at follow-up after 6–8 months, gender-specific risk factors for and clinical presentation of repeat infections.

**Methods and design:**

Sesam City is a drop-in clinic in the city centre of Stockholm. Patients 20 years and older are admitted. During 2007, the clinic had 15 000 visits, 60% made by men. In December 2007, a cohort study began, and data collection was finished in April 2009. A total of 2813 study participants aged 20–39 years were recruited. Data collection included an anonymous self-administered paper-and-pen questionnaire on sexual behaviour, reproductive health and history of Chlamydia, and condom use. Chlamydia tests were performed by self-sampled specimens, analyzed by the ProbeTec (Becton Dickinson) method, Ct-positive specimens also analyzed with a nvCT-specific method. Data from medical records were summarized in clinical report forms. Patients positive for Chlamydia were retested 4 weeks after treatment. Contact tracing covered sexual contacts during the last 12 months. At follow-up 6–8 months after inclusion, Chlamydia tests were performed, and a new questionnaire and CRF completed.

**Discussion:**

A STI-clinic-based prospective cohort study allowed us to survey 2813 adult patients. The collected data will provide gender-specific information on the occurrence of and risk for repeat Chlamydia infection, the occurrence of nvCT, and clinical data and information on sexual behaviour and reproductive health, risk-taking and condom use.

## Background

### Genital infection with Chlamydia trachomatis in Sweden

Genital infection with Chlamydia trachomatis is the most common notifiable sexually transmissible infection in Sweden, and included in the Act of Communicable Diseases. Testing and treatment is free of charge. Contact tracing is mandatory. National statistics are based on reports from physicians and counsellors. Records are identified with a code in order to protect the identity of the patient.

An increasing number of cases is reported to the national surveillance system since the late 1990's, and statistics is available at The Swedish Institute for Infectious Disease Control [1]. In 1997, 13 905 cases were reported (157 cases per 100 000 population), and in 2006 32 555 cases (360 per 100 000 population). More cases are reported among females than males. The overall majority is young: teenagers and adults < 30 years.

Changes in sexual behaviour contribute to the increase of Ct [2]. In late 2006, a new variant of Chlamydia trachomatis (nvCT) was identified in Sweden [3]. The mutant has a deletion in the cryptic plasmid, and escaped diagnostic laboratory methods widely used in Sweden. When laboratories changed their methods to be able to identify nvCT, a steep increase of reported cases occurred [4]. In 2007, 47 099 cases (513 per 100 000 population) were reported, interpreted as a catch-up effect [5]. However, the increase of Chlamydia cases continues also when nvCT is included in testing systems used all over Sweden. For 2008 42 000 (454 cases per 100 000) were reported.

### Risk factors for Chlamydia and repeat infections

Young age has since long been identified as the most important risk factor for Chlamydial infection, and international guidelines recommend regular testing of women aged <25 years old [6]. Furthermore, one previous episode of Chlamydia has been found to be a risk factor for a recurrent or new infection, often within a year, and for both sexes. This has been shown in population-based as well as in observational studies from STI clinics [7-15]. The reason for a positive test at follow-up may depend on when testing is performed, as the reason may be a persistent infection, a reinfection by untreated partner/partners, or a new infection. Repeat testing of Chlamydia-infected persons is recommended at different times: as a test of cure, after 3 months, annually, and according to the perceived risk of the patient.

The occurrence of repeat Chlamydial infection cannot easily be calculated on the basis of national statistics due to anonymization of records. However, a retrospective study of the anonymized STD registry was performed by Bengtsson in 2004 [16]. By use of Chlamydia statistics from 1997 to 2000, and maximum likelihood theory, an estimation of the risk for recurrent diagnoses was performed. The risk ratios vary between the sexes and different age groups, but increase during the investigated period. For 1999, 18% of teenage girls with Chlamydia were estimated to have a new infection within 2 years, compared with 10% of of teenage boys, and 8% of both sexes aged 25–29 years. Consequently, rescreening of individuals who have tested positive for Chlamydia is recommended, but further investigations and clinical studies have not been performed.

### Repeat infections and sequelae in women

The impact of repeat infections is the risk for further spread, but also an increased risk for Chlamydia-related complications. Women are most at risk, pelvic inflammatory disease (PID), ectopic pregnancy and infertility are well-known complications to Chlamydial infections. The risk for infertility has been shown to increase with repeat episodes of salpingitis. Today, the excess of female infertility after Chlamydial infections is questioned, and a few investigators have recently critically reviewed the basis of current knowledge [17-20]. It is a fact that the natural history of infection is not completely understood, and it is a paradox that parallel to the increase of cases fewer complications are observed by the clinicians. However, "silent salpingitis" is a clinical entity, and ectopic pregnancy is related to Chlamydia infections shown in register-based investigations from Sweden and Norway [21-23].

### Objectives and rationale for the study

The occurrence of repeat Chlamydial infection in Sweden is still to be investigated, as well as the clinical presentation of repeat infections. The challenge for STI-clinics and low-threshold clinics is awareness of symptoms and signs that indicate risk for complicated Chlamydial infection, and to treat it accordingly. The aim of the present prospective cohort study is to investigate the occurrence of repeat infections among the visitors to the Sesam City Clinic.

The four study questions are: after 6 – 8 months follow-up, do patients treated for Chlamydial infection have a higher rate of recurrency/repeat infection, than subjects testing negative for Chlamydia at inclusion? Is there a difference in clinical presentation between initial and repeat infections? Is there a difference in repeat infections in mutant (nvCT) and non-mutant (wCT) chlamydial infections? Can behavioural risk factors for repeat Chlamydial infection be identified?

The data collected will provide information for the planning of appropriate clinical care for STI-clinic visitors, with focus on Chlamydia and indications for retesting.

## Methods and design

The study is designed as a prospective cohort study with initial data collection at inclusion, and follow-up recording 6–8 months later. Inclusion criteria are age 20–39 years, knowledge of Swedish sufficient to answer the study questionnaires, and a sample for Chlamydia. The objective is both to compare Chlamydia infection status of the participants at inclusion and at the follow-up visit, and to investigate potential risk factors for infection and reinfection.

### Setting

In 2006, the Sesam City project began in order to facilitate Chlamydia screening for young adults. Sesam City is now a drop-in STI clinic for adults aged from 20 years and older in the city centre of Stockholm, run by the Dept. of Obstetrics and Gynaecology at the Karolinska University Hospital. It is open five days a week, in the afternoon and early evening, and is the only hospital-run clinic of its kind in the capital. The staff consists of specially trained nurses and midwives, counsellors and a part-time gynaecologist. Venereologists are recruited from the Dept. of Dermatovenereology from the Karolinska University Hospital.

In 2006, approx. 15 000 visits were made, 60% by men. The majority of all patients were tested for Chlamydia. Of all patients, 10% tested positive. In a pilot project from the clinic in early 2007, the new variant of Chlamydia, nvCT, represented 23% of 115 consecutively diagnosed Chlamydia cases [24].

The inclusion period with the recruitment of study participants started December 12 2007 and continued until June 2 2008. The follow-up period for data collection took place between August 22 2008 to April 30 2009. The data collection upon inclusion consisted of self administered paper-and-pen questionnaires, laboratory results for Chlamydia and separately for nvCT, the clinical data from the medical records summarized into clinical report forms (CRFs), and results of the contact tracing All Chlamydia positive patients were scheduled for a test of cure 4–6 weeks after finishing their treatment, and answered a one-page questionnaire at that time. At follow-up a new questionnaire was completed, a lab test for Chlamydia performed, and a CRF completed.

### Participants

All visitors to the clinic were consecutively invited to participate in the study according to the inclusion criteria. Information was provided at the reception desk by one of the clinic's specially trained nurses. Visitors who agreed to participate in the study were asked to answer the questionnaire, and to leave a sample for Chlamydia testing. Patients with genital symptoms were examined by a physician. All symptom-free patients were seen by a nurse. Six to eight months after the initial visit, all participants were invited to return for the follow-up visit. Due to the summer holiday period in Sweden during July and August, the follow-up period started in late August.

When patients arrived at Sesam City, they were met by a nurse at the clinic's reception desk. The desk is separated by a glass door from the common waiting room. Patients who would consider participation in the study received an envelope, marked with a study number, and a ballpoint pen. The envelope contained information on the study to be kept by the respondent, a consent form to sign, and a contact form with the patient's full ID and address to be used for the invitation to the follow-up visit. Patients could choose to be contacted via sms, e-mail, phone or post. The envelope also contained a ten page paper-and-pen questionnaire. The questionnaire did not contain any ID but a study participant number that was printed on top. (All questionnaires and CRFs in the study were marked with the study number only, to make all scanning and computer analyses anonymous.)

At the clinical visit, the patient was asked to return the envelope with its contents to the nurse or doctor, who could also answer questions concerning the study. The questionnaire, the consent form and the contact form for the follow-up visit remained in the envelope, and all envelopes were collected and opened by the study staff after each clinical session. The questionnaires were kept separately. The study number was matched to the patients ID on a list covering names and ID's for each clinical session. The "code list" was then kept in a separate secure, locked away register. Consent forms and follow-up visit forms were stored separately.

### Variables

The main variables are the results of the Chlamydia tests at inclusion and follow-up, related to a history of Chlamydia and to symptoms and signs in female respondents. The number of partners during the last 12 months and use of condoms are of particular interest. Some other variables may be of interest per se. In the questionnaire there are for example questions about what sources patients remember receiving information about Chlamydia from, which may be of interest when planning information campaigns. Another variable of interest is the patients' reports on sexual contacts to the counsellor performing the contact tracing, which may be compared with the number of contacts they previously reported in the questionnaire.

### Ethics

The research protocol was approved by the local ethical committee at the Karolinska University Hospital Solna, Stockholm, Sweden.

## Data collection at inclusion

### Questionnaire

The questionnaire (Additional file 1) used upon inclusion was self administered and comprised 10 pages, with multiple questions with fixed alternative answers. Age, sex and occupation, partner relations and sexual contact patterns and condom use was asked for, together with questions on the history of Chlamydia and Chlamydia testing, and other STIs. Use of alcohol and drugs was investigated, sexual abuse, use of contraception including emergency contraception, and abortion. Terms like "heterosexual", "homosexual" or "bisexual" were not used, respondents instead were asked for the gender of sexpartner/sexpartners. In a final question, the respondent was asked to accept or not accept to turn up for the follow-up visit.

At the development stage of the study the questionnaire was piloted, and gender-specific feedback and comments were taken into account when the questionnaire was completed. Appropriate ways to send an invitation for the follow-up visit were discussed as well.

### Laboratory

During the pilot period, a new method of Chlamydia sampling for females was introduced at the clinic, consisting of self-collected vaginal swabs put into first void urine and transported in the same plastic tube. Urine testing was already in use for men. The electronic referrals and test tubes were labelled "study patient" in order to separate, freeze and save samples. All samples were and brought daily to the Dept. of Microbiology at the Karolinska University Hospital.

Ct-analysis was performed by the ProbeTec™ (Becton-Dickinson [BD] Franklin Lanes, NJ, USA). All samples positive for Chlamydia were analyzed with a mutant-specific method, in order to differentiate between nvCT and wCT [3].

### Clinical routines

Patients with symptoms – discharge, dysuria, bleeding in females, discharge and dysuria in males – were examined by a physician and promptly treated according to clinical routines. Patients with a steady partner diagnosed with Chlamydia, or a referral letter on Chlamydia due to contact tracing. were treated promptly after sampling. According to indication, tests were taken for gonorrhoea, Mycoplasma genitalium, HIV and syphilis. Doctors were expected to examine a wet mount, and to conduct direct microscopy on stained specimens from urethra and cervix.

### Treatment of patients with Chlamydia

Treatment for Chlamydia was provided according to the Swedish routine with doxycycline 100 mg 10, 2 tablets at the same time the first day, followed by 1 tablet daily. Sexual abstinence during treatment, and participation in contact tracing, belongs to the routine.

All patients with a positive sample for Chlamydia met with a counsellor for contact tracing with regard to the last 12 months. The appointment was arranged without delay, and could take place the same day as treatment was provided. A visit for a "test of cure" with a new Chlamydia test was scheduled 4 weeks after finishing the treatment. This test of cure is specific for the study, and not part of the usual routine. Patients answered a one-page paper-and-pen questionnaire covering compliance, whether a regular partner had been treated, whether they had abstained from having sex with a partner during treatment, and whether they had had sex with a new partner after finishing treatment.

### The counsellors' data collection

Mandatory contact tracing is part of the Swedish routine for Chlamydia treatment. The contact tracing at Sesam City is carried out by specially trained counsellors. The counsellors met with all patients with Chlamydia, and collected information on sexual partners 12 months back in time. They contacted all partners, and as far as possible received information on the results of their Chlamydia tests, even if tested at other clinics. A few patients did not show up for contact tracing, and were after reminders referred to the County Medical Officer in Stockholm County for further handling. All information was summarized into a log in excel format by two of the counsellors.

### CRF

All respondents' medical records were summarized into clinical report forms, stating reason for visit, genital symptoms, if examined by a doctor or not, results from the genital examination and direct microscopy, whether treatment was provided before laboratory results, and results of all laboratory tests, and diagnoses. A CRF covered one printed page, and was answered by ticking boxes, filled out retrospectively.

### The study log

A log in excel format was kept by the chief investigator for keeping track on participation rates, results of Chlamydia tests including nvCT, treatment and follow-up after treatment for Chlamydia, tests of cure, and on further testing during the intermission between inclusion and late follow-up (interim visits), date for the 6–8 months follow-up, number of reminders, and the results of the follow-up Chlamydia tests.

## Data collection at follow-up

Patients were invited according to their wishes, i.e. through sms, e-mail, phone call or letter. They were welcome to the clinic within two weeks, at any opening hours and also early mornings. They were asked to answer a new paper-and-pen questionnaire and to perform a Chlamydia test. Condoms and Safe-sex information leaflets were generously distributed. A maximum of three reminders were used. After six weeks, and due to low follow-up-rates, we introduced a cinema ticket as a reward for participation. Treatment, contact tracing and a test-of-cure was performed for all subjects testing positive for Chlamydia during follow-up, in the same way as during the inclusion period. A one-page CRF was been completed by the chief investigator.

The follow-up questionnaire comprised 4 pages (Additional file 2), and contained questions concerning genital symptoms related to Chlamydia, sexual behaviour since the entry visit, and condom use. Two questions addressed Chlamydia: whether the respondent had been tested at another clinic since inclusion, and if so the outcome of the test.

### Bias

Patients testing positive for Chlamydia in the study have been offered a more intensive follow-up than standard routines in Sweden, as a "test of cure" has been performed a month after finishing medication. Together with an efficient partner notification provided by the counsellors, and treatment of partner/partners, the risk for persistent infection, or reinfections by an untreated partner, should be minimized. Thus, the study has included an intervention. It is also possible that the probability of drop-out at follow-up is related to variables of interest. If risk factors for drop-out are captured at study entry it can be investigated, while changes in lifestyle between entry and follow-up can not be determined.

### Study size

Based on previous research on repeat infections with Chlamydia [7-15], we estimated that we needed to recruit a total of 2552 patients to obtain a power of 80% with a type I error of 5%, assuming that 10% of the respondent included at entry would be Chlamydia positive, that 10% of them would be positive again at follow-up, and that 5% of the Ct-negative respondents at entry would be Ct-positive at follow-up. This would allow us to study differences in risk factors between those Ct-positive during both visits, and those first negative and subsequently positive at follow-up. To cover for an eventual 30% drop-out at follow-up, we decided to distribute 3500 questionnaires.

### Participation results

During the inclusion period December 12 2007 to June 2 2008, a total of 5 244 persons aged 20–39 years made visits at the Sesam City Clinic, 3067 (58.5%) men and 2177 (41.5%) women. A total of 3500 questionnaires were distributed, and 2813 consented to participation. The total response rate was 53.6% (2813/5244), 46.8% men (1435/3067) and 63.3% (1378/2177) women.

The Chlamydia test was an essential inclusion criterion. The frequency of positive tests among all study participants was 10,9% (306/2813), 12,8% (183/1435) among males and 8,9% (123/1378) among females.

## Discussion

High Chlamydial reinfection rates among teenagers and young adults are reported from US, UK and Norway. The relationship between "being infected" and "being infected again" is estimated only by mathematical models in Sweden, indicating high and increasing rates of recurrency [16]. The present study is the first attempt to investigate recurrent Chlamydial infections in a clinical setting, and is now performed as a prospective cohort study at a low threshold STI-clinic for adults in Stockholm.

A major issue when planning this study was to identify potential behavioural risk factors for recurrent or repeat Chlamydial infections, in order to find out if a history of Chlamydial infection and/or a positive test for Chlamydia at inclusion would indicate an increased risk for another positive test at follow-up.

Thorough contact tracing was performed, and "test of cure", in order to minimize early reinfections by untreated sexual partners. Identification of nvCT contributes to the understanding of the occurrence of nvCT investigated earlier at the clinic [24].

Furthermore, the study aims to describe the clinical presentation of Chlamydia, with special regard to women. It is wellknown that Chlamydia can cause salpingitis, and that salpingitis (also clinically asymptomatic or "silent") can cause tubal infertility and ectopic pregnancy [21-23]. Repeat infections increase this risk [25]. The clinical management of female "repeaters" is a challenge, salpingitis is a difficult clinical diagnosis, but a positive test for Chlamydia should include a clinical evaluation of whether the infection is complicated or uncomplicated, for prompt treatment.

Recommendations on repeat testing remain to be discussed.

## Competing interests

The authors declare that they have no competing interests.

## Authors' contributions

KE initiated and participated in the design of the project including clinical routines, and drafted the manuscript. SK-B participated in the design of the study and performed the initial statistical analyses. MB performed all the final statistical analyses. MRÖ was responsible for the microbiological analyses for Chlamydia trachomatis including the specific analyses for the new variant of Chlamydia trachomatis, nvCT. IQ and JE performed and reported all contact tracing. All authors read and approved the final manuscript.

## Pre-publication history

The pre-publication history for this paper can be accessed here:



## Supplementary Material

Additional file 1 **Questionnaire A.** Click here for file

Additional file 2 **Questionnaire B.** Click here for file
