# Supplementary material for: Repeat infection with Chlamydia trachomatis: a prospective cohort study from an STI-clinic in Stockholm
Source: BMC Public Health. 2009 Jun 22;9:198. doi: 10.1186/1471-2458-9-198 (PMC2709620; doi:10.1186/1471-2458-9-198)
Supplement: Additional file 1 — Questionnaire A. [file 1471-2458-9-198-S1.doc]

# Chlamydia study Sesam City 2007-2008

Questionnaire A, for inclusion

## A. Background

A1. Date (yy mm dd) [ ] [ ] [ ]

A2. What year where you born? 19 [ ]

A3. Sex?

[ ] Male

[ ] Female

A4. Is this your first visit to Sesam City?

[ ] Yes

[ ] No

A5. What is your main occupation?

[ ] Employed

[ ] Studying

[ ] Unemployed

[ ] Long-term sick leave

[ ] Other, _______________

A6. Are you …

[ ] Married

[ ] Live-in boy/girl friend

[ ] Partner (not living together)

[ ] Single

[ ] Other, _______________

A7. Do you have children?

[ ] Yes

[ ] No

## B. A few questions concerning Chlamydia

B1. Why do you wish a test for Chlamydia? You may tick several boxes

[ ] Have had sex with casual partner in Sweden

[ ] Have had sex with casual partner abroad

[ ] Experienced symptoms

[ ] New partner has asked me to check

[ ] A partner has Chlamydia

[ ] Received a contact tracing letter concerning Chlamydia

[ ] For safety

[ ] Other, ______________

B2a. Have you previously been tested for Chlamydia?

[ ] Never (Go to B4)

[ ] Once

[ ] 2-3 times

[ ] 4 or more times

[ ] Don’t know

B2b. Have you been tested during the last 12 months?

[ ] Yes

[ ] No

[ ] Don’t remember

B2c. When tested for Chlamydia have you been advised about safer sex, condoms etc?

[ ] Yes

[ ] No

[ ] Don’t remember

B3a. Have you ever had Chlamydia?

[ ] Never (Go to B4)

[ ] Once

[ ] 2-3 times

[ ] 4 or more times

[ ] Don’t know/don’t remember

B3b. Have you had Chlamydia during the last 12 months?

[ ] Yes

[ ] No

[ ] Don’t remember

B3c. When treated for Chlamydia have you been advised about safer sex, condoms etc?

[ ] Yes

[ ] No

[ ] Don’t remember

B4. Have you received information concerning Chlamydia in any of the follow ways? You may tick several boxes

[ ] Internet

[ ] Information in school

[ ] Youth clinic

[ ] Brochures

[ ] Through friends

[ ] From partner

[ ] Parents

[ ] Siblings

[ ] Newspapers

[ ] Television

[ ] From doctor or nurse, not at a youth clinic

[ ] Posters around town

[ ] Never heard of Chlamydia

## C. A few questions concerning other STDs

C1. Have you ever been tested for HIV?

[ ] Never

[ ] Once

[ ] 2-3 times

[ ] 4 or more times

[ ] Don’t know/don’t remember

C2. Have you ever been tested for, or had… Answer each question.

Yes No Don’t know

1 Genital herpes [ ] [ ] [ ]

2 Condylomas [ ] [ ] [ ]

3 Gonorrhoea [ ] [ ] [ ]

4 Mycoplasma genitalium [ ] [ ] [ ]

5 Unspecific urethritis [ ] [ ] [ ]

6 Syphilis [ ] [ ] [ ]

7 Hepatitis B [ ] [ ] [ ]

8 HIV [ ] [ ] [ ]

## D. Questions about your current sexual situation

D1a. Are you in a relationship? (Answer each question)

Yes, for how long?

No <1 month 6-12 months >12 months

1 With a man [ ] [ ] [ ] [ ]

2 With a woman [ ] [ ] [ ] [ ]

3 With several people [ ] [ ] [ ] [ ]

If no on all questions, go to D2

D1b. Have you had any sexual relations outside your current relationship during the last 12 months?

[ ] No

[ ]Yes, once

[ ] Yes, 2-3 times

[ ] Yes, 4 or more times

[ ] Yes, with another partner I’ve met several times

[ ] Don’t know/don’t remember

D2. How or where have you met new or casual partners during the last 12 months?

Choose a maximum of three alternatives (most relevant).

[ ] Through the workplace

[ ] Through friends

[ ] In a nightclub/disco

[ ] In a restaurant/Café

[ ] In school

[ ] Through the Internet

[ ] On holyday abroad

[ ] Trough work/studies abroad

[ ] At a conference/office party

[ ] Other

[ ] Have not meet a new or temporary partner during the last 12 months (Go to D5)

D3. How many people have you had sexual relations with during the last 12 months?

Approximate if you don’t remember.

Total number of people [ ][ ][ ]

Number of men [ ][ ][ ]

Number of women [ ][ ][ ]

D4. How many of these were casual partners, i.e. someone you only had sex with a limited number of times?

Total number of people [ ][ ][ ]

Number of men [ ][ ][ ]

Number of women [ ][ ][ ]

D5. When was your last sexual encounter?

[ ] During the last 7 days

[ ] 1-4 weeks ago

[ ] 1-3 months ago

[ ] 4-6 months ago

[ ] 7-12 months ago

[ ] More than 12 months ago

[ ] Don’t remember

D6. With whom was your last sexual contact?

Male Female

Steady partner [ ] [ ]

(Husband/wife, boy/girl friend, live-in boy/girl friend, registered partner)

2 Recurring partner [ ] [ ]

(Someone you’ve had previous sexual relations with but are not in a steady relationship with)

3 Temporary known sexual contact [ ] [ ]

(Someone you know but has never had a previous sexual relationship with)

4 Temporary unknown sexual contact [ ] [ ]

(Someone you’ve never met before)

5 Several people at once [ ] [ ]

(Three way, group sex)

6 Other type of partner ________________

D7. What type of sex did you have during your last sexual contact? Also answer of you used a condom. (Answer all questions)

Type of Sex Did you use a condom?

Yes, during the entire act - Yes, during parts of the act - No

Vaginal intercourse [ ] [ ] [ ] [ ]

Anal intercourse [ ] [ ] [ ] [ ]

Oral sex [ ] [ ] [ ] [ ]

Pettin [ ] [ ] [ ] [ ]

Other [ ] [ ] [ ] [ ]

D8. Have you ever been forced into a sexual act against your will? You may tick several boxes

[ ] Yes, as a child

[ ] Yes, as a teenager

[ ] Yes, as an adult

[ ] No

### E. Questions about condoms

E1. Do you use a condom with new or temporary partners?

[ ] Always

[ ] Often

[ ] Rarely

[ ] Never

E2. Is getting hold of condoms a problem for you? You may tick several boxes

[ ] I never get condoms

[ ] No, it is ok

[ ] Yes, it is embarrassing

[ ] Yes, it is expensive

[ ] Yes, because of ______

[ ] I never use condoms

E3. Do you take responsibility to get condoms?

[ ] Always

[ ] Sometimes

[ ] Rarely

[ ] Never

[ ] I never use condoms

E4. Have you ever experienced a condom breaking or falling off during intercourse?

[ ] Often

[ ] Occasionally

[ ] Once or twice

[ ] Don’t remember

[ ] I never use condoms

E5. Have you ever experienced that you or your partner lost erection while using a condom?

[ ] Often

[ ] Occasionally

[ ] Once or twice

[ ] Never

[ ] Don’t remember

[ ] I never use condoms

E6. Have you ever experienced any of the following problems that have led you to not use a condom?

[ ] Didn’t plan for sex, did not bring a condom

[ ] Feel less intimate with my partner

[ ] Disturbed by the interruption when putting the condom on

[ ] Decrease of sexual arousal

[ ] I have a hard time reaching orgasm/ejaculating when using a condom

[ ] Partner has a hard time reaching orgasm/ejaculating when using a condom

[ ] Other _____________

## F. Questions about alcohol and drugs

F1a. Have you during the last 6 months consumed alcohol before sex?

[ ] Yes, once

[ ] Yes, occasionally

[ ] No (Go to F2a)

[ ] Don’t know/don’t remember (Go to F2a)

F1b. Do you feel that alcohol influenced you to take greater sexual risks compared to normal?

[ ] Yes, a great deal of influence

[ ] Yes, some influence

[ ] Yes, but very little influence

[ ] No, no influence at all

[ ] Don’t know/don’t remember

F2a. Have you during the last 6 months taken any other drugs than alcohol I connected to sex?

[ ] Yes, once

[ ] Yes, on several occasions

[ ] No (Go to G1 or H1)

[ ] Don’t know/don’t remember (Go to G1 or H1)

F2b. Which drug? Answer all questions

Yes No

Cannabis [ ] [ ]

Amphetamine [ ] [ ]

Cocaine [ ] [ ]

GHB [ ] [ ]

Ecstasy [ ] [ ]

Heroine [ ] [ ]

Other [ ] [ ] _____________

Don’t know/don’t

remember [ ] [ ]

F2c. Do you feel that drugs influenced you to take greater sexual risks compared to normal?

[ ] Yes, a great deal of influence

[ ] Yes, some influence

[ ] Yes, but very little influence

[ ] No, no influence at all

[ ] Don’t know/don’t remember

## G. Only for men

G1. Have you ever made a partner pregnant without intending to do so?

[ ] Yes, once

[ ] Yes, several times

[ ] No

[ ] Don’t know/don’t remember

Go to S1

## H. Questions on contraceptives, for women only

H1. What contraceptive(s) are you currently using? (Answer all questions)

Yes No

Combined oral contraceptive pill [ ] [ ]

Progesterone only pill [ ] [ ]

Contraceptive implant [ ] [ ]

Contraceptive vaginal ring [ ] [ ]

Intrauterine device [ ] [ ]

Diaphragm [ ] [ ]

Condom [ ] [ ]

Interrupted intercourse/safe periods [ ] [ ]

Sterilized [ ] [ ]

No contraceptive, want to become pregnant [ ] [ ]

No contraceptive, other reason [ ] [ ] reason: ________________

H2. Are you happy with your method?

[ ] Yes

[ ] No

H3. Have you ever used emergency contraception (the morning after pill)?

[ ] Yes, once

[ ] Yes, several times

[ ] No

H4. Have you ever had an abortion?

[ ] Yes, once

[ ] Yes, several times

[ ] No

## S. Finally

S1. The study is based on that you leave a Chlamydia test in six months and fill in a short questioner. We will call you for an appointment in the manner you prefer, - SMS, email, phone call, letter.

Do you agree to a return visit?

[ ] Yes

[ ] No

If you agree, please fill in the form concerning how you wish to be contacted in connection to your return visit.

THANK YOU FOR YOUR ASSISTANCE!

# Author: Karin Edgardh
