# Supplementary material for: Repeat infection with Chlamydia trachomatis: a prospective cohort study from an STI-clinic in Stockholm
Source: BMC Public Health. 2009 Jun 22;9:198. doi: 10.1186/1471-2458-9-198 (PMC2709620; doi:10.1186/1471-2458-9-198)
Supplement: Additional file 2 — Questionnaire B. [file 1471-2458-9-198-S2.doc]

# Chlamydia study Sesam City 2007-2008, Questionnaire B, for follow-up

# A. Background

A1. Today’s date [ ][ ] [ ][ ] [ ][ ]

A2. Are you currently experiencing any problems that you need to book an appointment to get examined?

[ ] Yes

[ ] No

A3. Do you suspect that you have an STD?

[ ] Yes

[ ] No

# B. Questions concerning your current sexual situation

**All the following questions concern the time after the first visit in the Chlamydia study at Sesam City. For most participants this means during the last 6-8 months.**

B1. Are you currently in a relationship? (answer all questions)

No Yes, for how long?

<1 month 1–5 months 6-12 months >12 months

With a man

[ ] [ ] [ ] [ ] [ ]

With a woman

[ ] [ ] [ ] [ ] [ ]

With several people simultaneously

[ ] [ ] [ ] [ ] [ ]

B2. With how many people have you had sexual intercourse with during the last 6 months? Estimate if you don’t remember the exact number

Total [ ][ ][ ]

Men [ ][ ][ ]

Women [ ][ ][ ]

B3. How many of these where casual partners, i.e. someone you had sex with only once?

Total [ ][ ][ ]

Men [ ][ ][ ]

Women [ ][ ][ ]

B4. When did you last have sex with someone?

[ ] During the last 7 days

[ ]1-4 weeks ago

[ ]1-3 months ago

[ ] 4-6 months ago

[ ] More then 6 months ago

[ ] Don’t remember

B5. Who did you have sex with the last time you had sex?

Yes Male Female

Steady partner [ ] [ ] [ ]

Reoccurring casual partner [ ] [ ] [ ]

(someone you have had sex with before but are not involved with in a steady relationship)

Casual known partner [ ] [ ] [ ]

Casual unknown partner [ ] [ ] [ ]

Several partners at once [ ] [ ] [ ]

(group sex)

Other kind of partner [ ] _____________________

B6. What type of sex did you have during your last sexual contact? Also answer of you used a condom. (Answer all questions)

Type of Sex Did you use a condom?

Yes, entire act - Yes, parts of the act - No

Vaginal intercourse [ ] [ ] [ ] [ ]

Anal intercourse [ ] [ ] [ ] [ ]

Oral sex [ ] [ ] [ ] [ ]

Petting [ ] [ ] [ ] [ ]

Other [ ] [ ] [ ] [ ]

B7. Have you had sex while traveling abroad, with a partner you met during your travels?

[ ] Yes

[ ] No

B8. How or where have you met new or casual partners? (Choose a maximum of three alternatives)

[ ] Through the workplace

[ ] Through friends

[ ] In a nightclub/disco

[ ] In a restaurant/Café

[ ] In school

[ ] Through the Internet

[ ] On holyday abroad

[ ] Trough work/studies abroad

[ ] At a conference/office party

[ ] Other

[ ] Have not met a new or temporary partner during the last 6 months

# C. A few questions concerning condoms

**All the following questions concern the time after the first visit when your joined the Chlamydia study at Sesam City. For most participants this means during the last 6-8 months.**

C1. Have you used condoms with new sexual contacts?

[ ] Always

[ ] Often

[ ] Rarely

[ ] Never

[ ] No new sexual partners

C2. Have you used condoms with casual sexual contacts?

[ ] Always

[ ] Often

[ ] Rarely

[ ] Never

[ ] No new sexual partners

C3. Did you use a condom during your last vaginal intercourse?

[ ] Yes

[ ] No

[ ] Have not had vaginal intercourse

C4. Did you use a condom during your last anal intercourse?

[ ] Yes

[ ] No

[ ] Have not had anal intercourse

# D. A few questions concerning Chlamydia

**All the following questions concern the time after the first visit in the Chlamydia study at Sesam City. For most participants this means during the last 6-8 months.**

D1. Have you had a test for Chlamydia since you enlisted for this Chlamydia study?

[ ] Once

[ ] More then once

[ ] No (Go to D2)

[ ] Don’t know/ don’t remember (Go to D2)

D1a. If yes, did you have Chlamydia?

[ ] Yes

[ ] No

[ ] Don’t know/don’t remember

# E. One questions concerning alcohol

E1. Do you feel that alcohol influenced you to take greater sexual risks compared to normal?

[ ] Yes, a great deal of influence

[ ] Yes, some influence

[ ] Yes, but very little influence

[ ] No, no influence at all

[ ] Don’t know/don’t remember

# F. Question Woman

F1. Have you used emergency contraceptive (the morning after pill) since you enlisted for this Chlamydia study at Sesam City?

[ ] Yes, once

[ ] Yes, several times

[ ] No

[ ] Don’t know/don’t remember

# G. Questions about your visits to Sesam City

G1. Have you during your visit(s) to Sesam City received information…

About Chlamydia

[ ] Yes, good information [ ] Yes, but not enough [ ] No, no information at all [ ] No, had no need for such information

[ ]Don’t know/don’t remember

About safer sex, condoms and more

[ ] Yes, good information [ ] Yes, but not enough [ ] No, no information at all [ ] No, had no need for such information

[ ]Don’t know/don’t remember

About contraception

[ ] Yes, good information [ ] Yes, but not enough [ ] No, no information at all [ ] No, had no need for such information

[ ]Don’t know/don’t remember

G2. Are you interested about being able to book a new appointment for a talk concerning how you could decrease the risk for Chlamydia and other STDs?

[ ] Yes

[ ] No

[ ] Don’t know/don’t remember

G3. Are you interested about being able to book a new appointment for a talk concerning how you could decrease the risk for unwanted pregnancy?

[ ] Yes

[ ] No

[ ] Don’t know/don’t remember

G4. Other comments – please feel free to write your own comments.

# Author: Karin Edgardh
